# Ferrara Rings for Visual Rehabilitation in Eyes with Keratoconus and Previous Cross-Linking Using the Ferrara Ring Nomogram

**DOI:** 10.3390/vision5040045

**Published:** 2021-09-29

**Authors:** Cameron A. McLintock, James McKelvie, Ye Li, Samer Hamada, Damian Lake

**Affiliations:** 1 Corneoplastic Unit and Eye Bank, Queen Victoria Hospital, NHS Trust, East Grinstead, West Sussex RH19 3DZ, UK; cameronmclintock@hotmail.com (C.A.M.); samer.hamada@qvh.nhs.uk (S.H.); damian@damianlake.com (D.L.); 2 Department of Ophthalmology, Princess Alexandra Hospital, Brisbane, QLD 4102, Australia; li.ye1210@gmail.com; 3 Faculty of Medicine, University of Queensland, Brisbane, QLD 4006, Australia; 4 Department of Ophthalmology, Faculty of Medical and Health Sciences, University of Auckland, 85 Park Road, Grafton, Auckland 1023, New Zealand

**Keywords:** intrastromal corneal ring segments, Ferrara ring, keratoconus, corneal rings

## Abstract

Purpose: To report the visual, refractive and tomographic outcomes following the implantation of intrastromal corneal ring segments (ICRS) (Ferrara rings, AJL Ophthalmics, Miñano, Spain) in eyes with a history of keratoconus and corneal cross-linking using the Ferrara ring nomogram. Methods: Retrospective, interventional case series performed at the Corneoplastics Unit, Queen Victoria Hospital, East Grinstead, United Kingdom. Results: 21 eyes of 19 patients with a history of keratoconus and prior corneal collagen cross-linking had Ferrara Intrastromal Corneal Ring Segments implanted between December 2015 and October 2017. The number, thickness and length of ring segments was chosen based on the Ferrara ring company nomogram. Mean uncorrected visual acuity (UDVA) improved from 0.88 to 0.52 logMAR (*p* < 0.001). Mean corrected visual acuity (CDVA) improved from 0.47 to 0.36 logMAR (*p* = 0.046). The percentage of eyes achieving 20/40 UDVA and CDVA increased from 5% to 38% and from 38% to 67%, respectively. Of the eyes, 52.3% gained at least two lines of CDVA. The spherical equivalent improved from −7.51D to −3.76D (*p* < 0.001) and the refractive astigmatism magnitude improved from 5.14D to 2.76D (*p* = 0.004). There were significant improvements in the corneal tomography with mean keratometry (K_M_) improving from 50.40D (3.53) to 48.24D (3.00) (*p* = 0.01) and keratometric astigmatism magnitude improving from 5.14D (2.91) to 2.76D (1.67) (*p* = 0.004). Conclusion: Insertion of Ferrara rings in keratoconic eyes with a history of prior cross-linking using the company nomogram results in significant improvements in visual, refractive and tomographic outcomes.

## 1. Introduction

Keratoconus is a condition characterised by abnormal posterior corneal ectasia, an abnormal corneal thickness distribution, and clinical noninflammatory corneal thinning [1]. It results in corneal protrusion, irregular astigmatism, and decreased vision [2]. Contact lenses are the mainstay of visual rehabilitation in keratoconus, often resulting in excellent vision. However, contact lenses are not always tolerated by patients and, in such cases, surgical treatment may be required for visual rehabilitation. Surgical options for keratoconus include corneal transplantation, topography-guided photorefractive keratectomy (PRK), trans-epithelial phototherapeutic keratectomy (PTK), phakic intraocular lenses, intrastromal corneal ring segments (ICRS), or a combination of the above [3].

Intrastromal corneal ring segments are made from polymethyl–methacrylate (PMMA) and are inserted into the corneal stroma to flatten and regularise the cornea [4]. Initially used for myopia, their use was extended to eyes with keratoconus after researchers noted their ability to regularize tissue asymmetry [5]. Thus, not only are corneal rings able to reduce the myopia and regular astigmatism present in keratoconic corneas, but they can also reduce irregular astigmatism and its associated higher-order aberrations such as vertical coma [6]. Several types of intrastromal corneal ring segments exist. These include Ferrara rings (AJL Ophthalmics, Miñano, Spain), INTACS (Addition Technology, Inc., Fremont, CA, USA), KeraRings (Mediphacos, Belo Horizonte, Brazil), Corneal Ring (Visiontech, Belo Horizonte, Brazil), and Myorings (Dioptex GmbH, Linz, Austria) [6]. They vary according to their diameter, thickness, arc length, and cross-sectional shape. Ferrara rings have a triangular cross-sectional shape, with each segment having an internal diameter of 4.40 mm and an external diameter of 5.60 mm. The thickness of Ferrara rings varies from 150 to 350 microns in 50-micron increments [7].

Various nomograms have been developed to guide surgeons in their choice of ICRS. These nomograms aim to induce a particular change in corneal profile based on pre-operative parameters. For example, some authors have suggested a single ring for cases of inferior keratoconus and two segments for central cones, whereas others have based the choice of ring on the degree of myopic spherical equivalent [8,9,10,11]. Others still have based their ICRS choice on the degree of corneal astigmatism [12]. For surgeons implanting Ferrara rings, the manufacturer has developed an online nomogram with the aim of optimising patient outcomes. With this nomogram, the surgeon must provide the following information based on corneal tomography: flat and steep keratometry (K1 and K2) power and axis, corneal thickness of the thinnest point of the proposed ring track (5 mm diameter), corneal thickness at the steep axis at a 5 mm zone, and a description of the corneal shape as either an oval cone, a nipple cone or pellucid marginal degeneration. The nomogram then recommends to the surgeon how many rings and the thickness, arc length, and the depth at which the rings should be inserted.

To date, the only study which, to the authors’ knowledge, has reported outcomes of Ferrara ring insertion with the assistance of the company nomogram, excluded eyes with a history of cross-linking [13]. Given that evidence exists suggesting that the effect of ICRS implantation may be diminished in previously cross-linked eyes, the outcomes of the Ferrara nomogram in eyes with prior cross-linking remains unevaluated [14,15].

The present study aims to, for the first time, report the visual, refractive, and tomographic outcomes in eyes with keratoconus with a history of prior cross-linking following implantation of Ferrara rings using the company nomogram.

## 2. Materials and Methods

A retrospective interventional case series was performed through the Corneoplastics Unit, Queen Victoria Hospital, East Grinstead, United Kingdom. Patients with keratoconus were offered Ferrara ring implantation for visual rehabilitation if the following inclusion criteria were met: contact lens intolerance, unsatisfactory vision with spectacle correction, a history of corneal cross-linking at least 6 months prior, absence of significant central corneal scarring, and a corneal thickness at least 400 microns in the proposed ICRS tract.

Pre-operatively, the following demographic data were collected: age, sex, and eye laterality. The pre-operative visual and refractive data included subjective refraction, unaided distance visual acuity (UDVA), and corrected distance visual acuity (CDVA). All patients had corneal tomography performed with a pentacam (Oculus Gmbh, Wetzlar, Germany), from which the following data were collected for both the anterior and posterior corneal surfaces: flat (K1) and steep (K2) keratometry, and corneal astigmatism at the 3 mm zone (Astig). The maximum keratometry value (K_MAX_), Q value, pupil diameter, and total corneal lower and higher order aberrations (HOAs) were also collected.

The number of rings, ring thickness, arc length and incision site were based on the online company nomogram (ajlsa.com/nomograma). The following data were entered into the online nomogram for each patient, based on the pentacam corneal tomography: flat and steep keratometry (K1 and K2) power and axis, corneal thickness of the thinnest point of the proposed ring track (5 mm diameter), corneal thickness at the steep axis at a 5 mm zone, and a description of the corneal shape as either an oval cone, a nipple cone or pellucid marginal degeneration.

ICRS insertion was performed under topical anaesthesia (oxybuprocaine 0.4%). The centre of the cornea was marked with a marking pen at the operating microscope. The ICRS tunnels were created with the Ziemer Z6 femtosecond laser (Ziemer Ophthalmic Systems, Port, Switzerland). The depth of the tunnel was selected to be 80% of the thickness of the thinnest point of the cornea in the proposed tunnel tract. The rings were then inserted and advanced so that the tip of the ICRS was completely buried within the tunnel and there was no gape of the wound. Post-operatively, patients used ofloxacin 0.3% four times per day for one week. Patients were seen one day, one week, one month, and three months post-operatively. The same visual, refractive, and tomographic data that were collected at baseline were collected at follow-up appointments.

The visual and refractive outcomes were analysed using the standard graphs for reporting outcomes of refractive surgery [16,17,18]. Efficacy was assessed by determining the percentage of eyes achieving UDVA values of 20/40, the number of eyes achieving CDVA of 20/40, and the percentage of eyes achieving a post-operative UDVA equal to that of the pre-operative CDVA. Safety was assessed by the presence of any complications.

As Shapiro–Wilk significance testing showed that the data were not normally distributed, nonparametric tests were used to compare groups. Significance testing within groups was performed with a Wilcoxon signed-rank test, whereas significance tests between groups were completed using the independent two-group Mann–Whitney U test. Statistical analysis was completed using R statistical software (Foundation of Statistical Computing, Vienna, Austria).

All procedures were followed in accordance with the ethical standards of the responsible committee on human experimentation (institutional and national) and with the Helsinki Declaration of 1964, as revised in 2013. All patients provided their written consent prior to surgery and the principles of the Declaration of Helsinki were fully respected. The local IRB committee approved this study. It was not registered as a clinical trial because this was not required by the ethics committee given its retrospective nature.

## 3. Results

Table 1 shows the baseline demographic characteristics of this patient cohort. A total of 21 eyes of 19 patients were included in the study, 15 of whom were male and 4 were female. Based on the Amsler–Krumeich classification of keratoconus, 62% had grade one, 14% had grade two, 14% had grade three, and 10% had grade four disease. All eyes had at least 3 months follow-up (mean 4.1 months). Ten eyes had one ring segment inserted and eleven eyes had two segments inserted. The thickness of the ring segments ranged from 150 to 350 microns. The arc length of the ring segments ranged from 140 to 210 degrees. The ring segment number, thickness and arc length was determined by the online nomogram.

**Figure 1:**
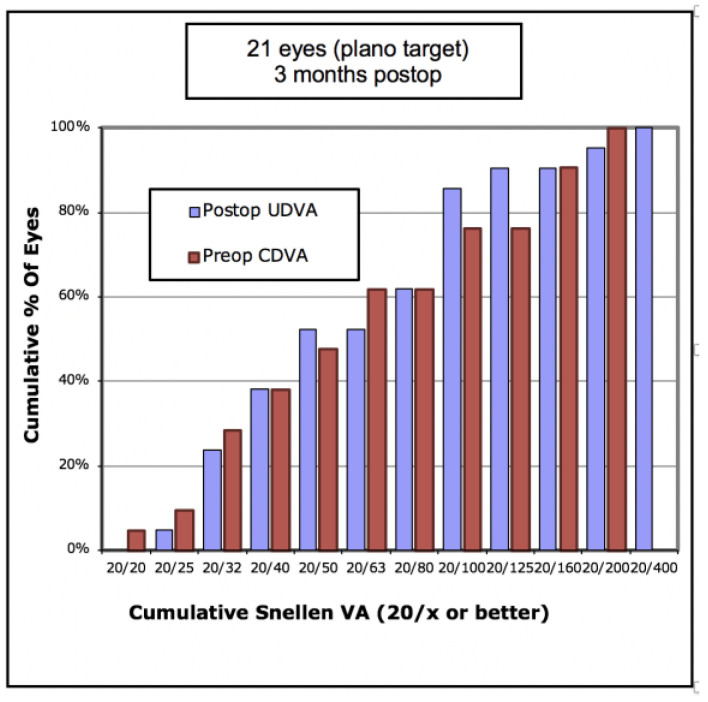
Cumulative pre-operative corrected distance visual acuity (CDVA) versus post-operative unaided distance visual acuity (UDVA) in 21 keratoconic eyes implanted with Ferrara intrastromal corneal ring segments.

**Figure 2:**
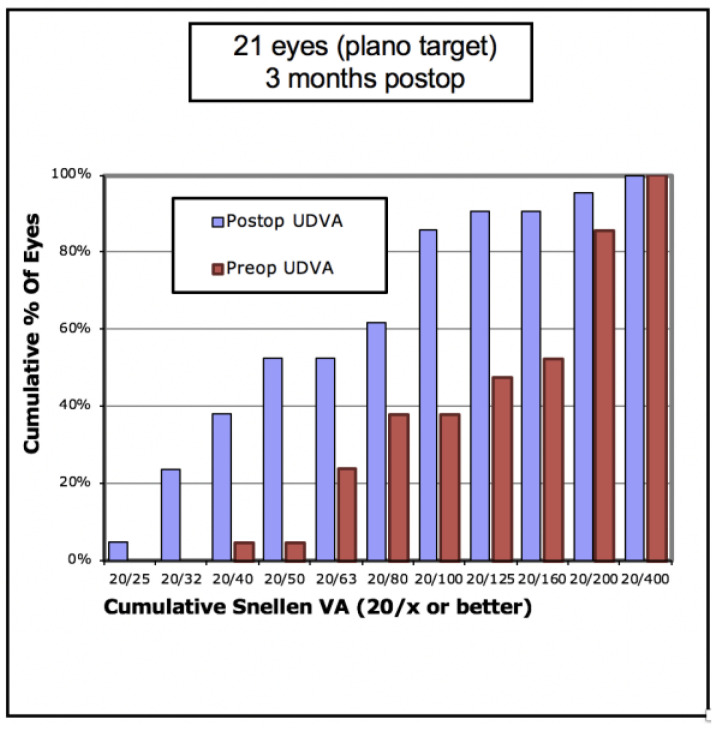
Cumulative pre-operative versus post-operative unaided distance visual acuity (UDVA) in 21 keratoconic eyes with Ferrara intrastromal corneal ring segments.

**Figure 3:**
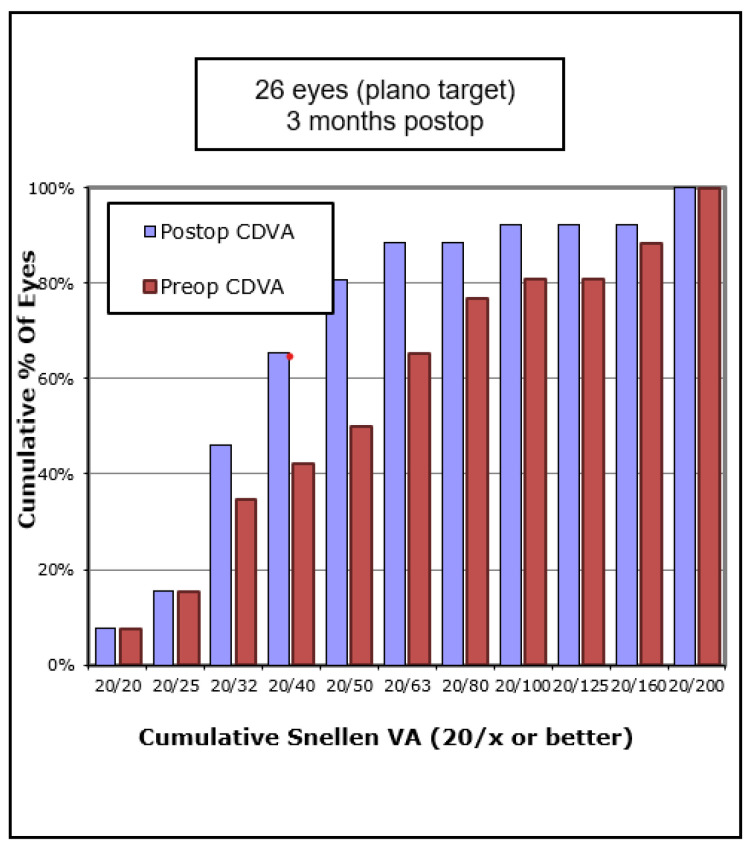
Cumulative pre-operative versus post-operative corrected distance visual acuity (CDVA) in 21 keratoconic eyes implanted with Ferrara intrastromal corneal ring segments.

**Figure 4:**
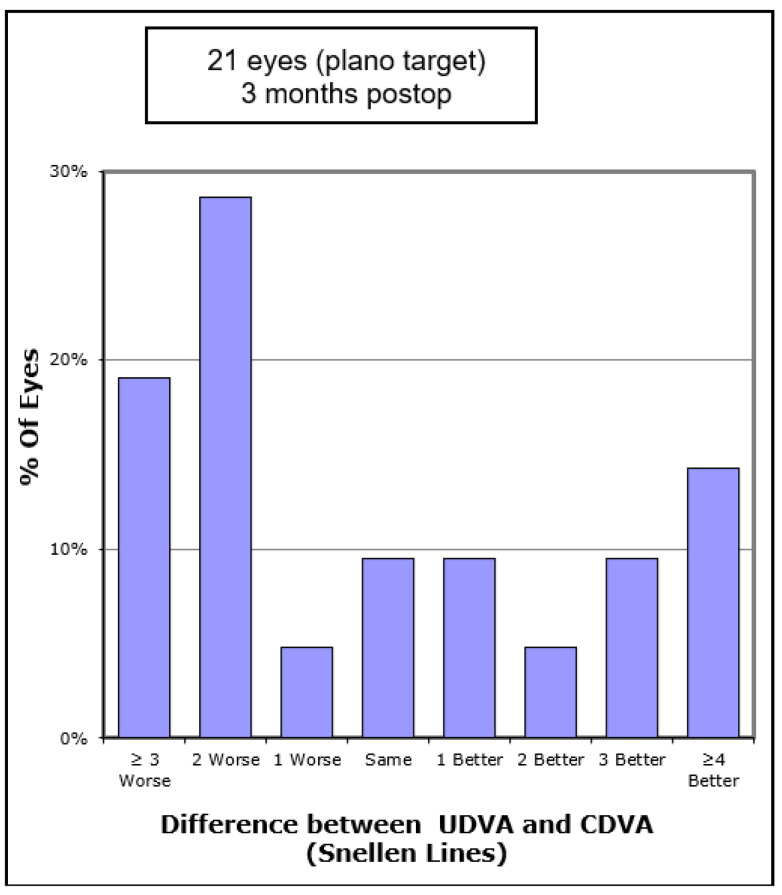
Number of Snellen lines difference between pre-operative CDVA and post-operative UDVA in 21 keratoconic eyes implanted with Ferrara intrastromal corneal ring segments.

**Figure 5:**
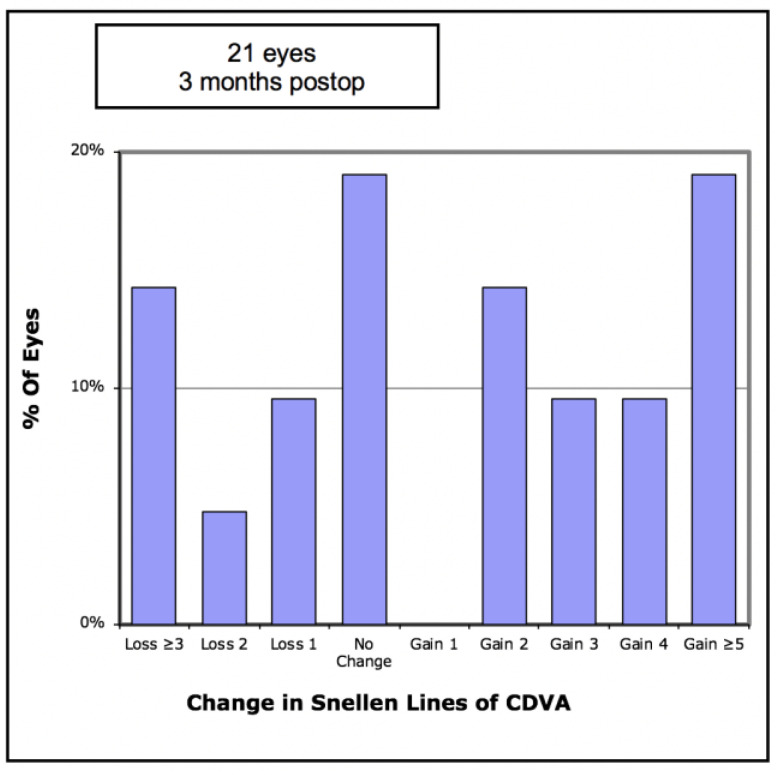
Number of Snellen lines difference between pre-operative and post-operative corrected distance visual acuity (CDVA) in 21 keratoconic eyes implanted with Ferrara intrastromal corneal ring segments.

In terms of visual outcomes, Table 2 shows there was a significant improvement in both mean UDVA and mean CDVA. Mean logMAR UDVA improved from 0.88 to 0.52 (*p* < 0.001) and mean CDVA improved from 0.47 to 0.36 (*p* = 0.042). Figure 1 shows cumulative post-operative UDVA versus pre-operative CDVA and, overall, a good agreement between these parameters is seen. Figure 2 shows that the percentage of eyes achieving 20/40 UDVA increased from 5% to 38% with ICRS insertion. Of the eyes, 47% achieved a post-operative UDVA equal to or better than the pre-operative CDVA (Figure 3). The percentage of eyes with 20/40 or better CDVA increased from 38% of eyes to 67% (Figure 4). Of the eyes, 52.3% gained CDVA, with 38% gaining three or more lines (Figure 5).

**Figure 6:**
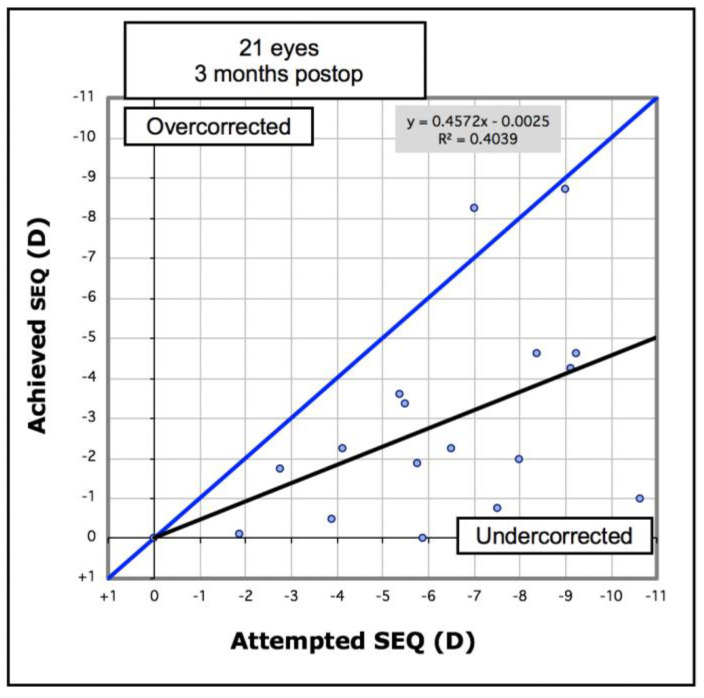
Attempted versus achieved spherical equivalent (SEQ) in 21 keratoconic eyes implanted with Ferrara intrastromal corneal ring segments. Black line = regression equation.

**Figure 7:**
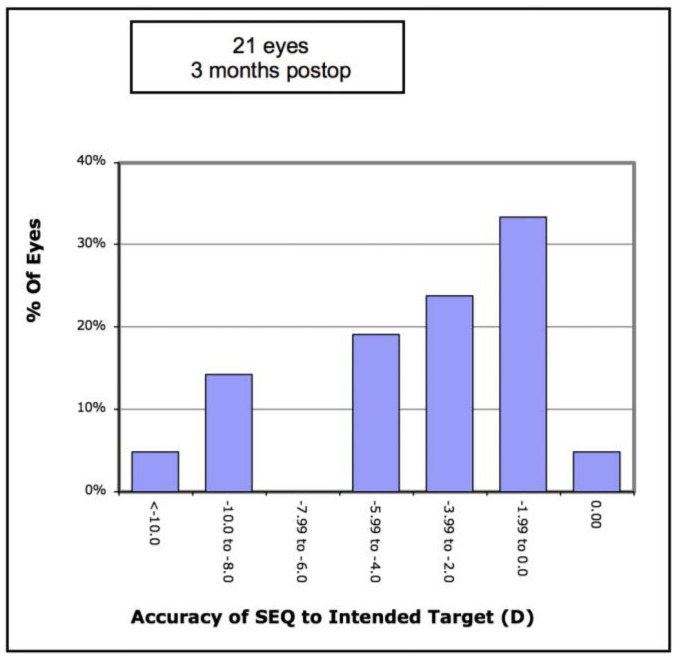
Accuracy of attempted versus achieved spherical equivalent (SEQ) in 21 keratoconic eyes implanted with Ferrara corneal ring segments.

**Figure 8:**
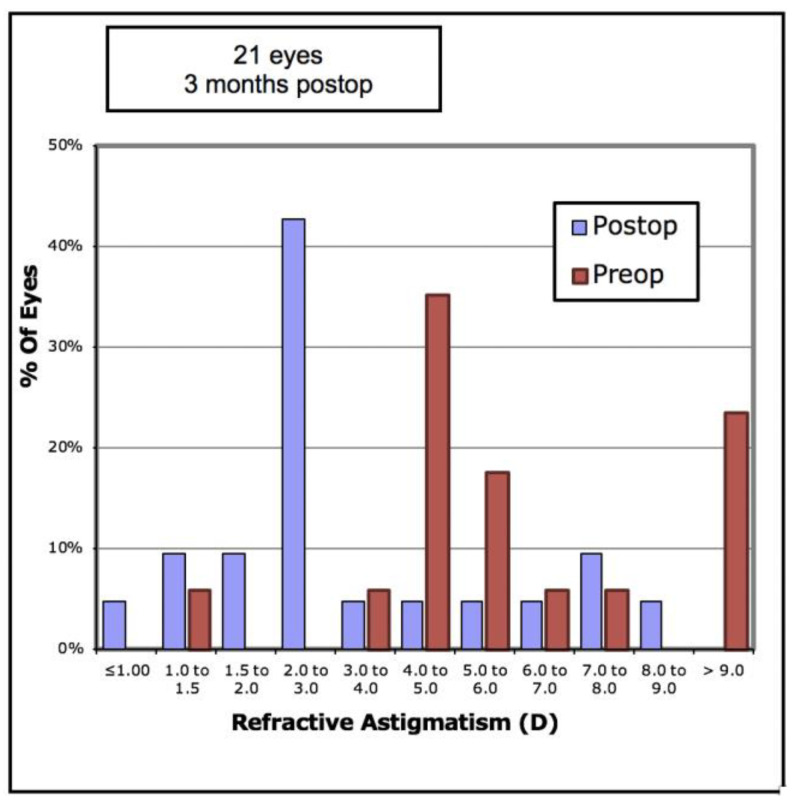
Pre-operative versus post-operative refractive astigmatism in 21 keratoconic eyes implanted with Ferrara intrastromal corneal ring segments.

Analysis of refractive outcomes showed a significant reduction in spherical equivalent from −7.51D to −3.76D (*p* < 0.001). Although there was a significant reduction in the degree of myopia in most cases, Figure 6 shows there was an undercorrection of myopia, which increased with the degree of myopia. Of the eyes, 33% achieved an SE of less than −2.0D, and 57% achieved an SE of less than −4.0D (Figure 7). The mean refractive astigmatism magnitude was reduced from 5.72D to 3.62D (*p* < 0.001). Figure 8 shows the pre-operative versus post-operative refractive astigmatism magnitude.

Table 3 shows the tomographic changes induced by the intrastromal corneal ring segments. There was a significant reduction in K_M_, K_MAX_ and keratometric astigmatism. There was also a significant improvement in Q values and total corneal higher-order aberrations.

There were no intra-operative or post-operative complications.

## 4. Discussion

Visual rehabilitation in eyes with keratoconus and contact lens intolerance is challenging. Traditionally, the treatment in such cases has been corneal transplantation. Although corneal grafts have a long and successful history of visual rehabilitation in keratoconus, corneal grafts are not without their difficulties. Compared to ICRS insertion, corneal transplantation is more invasive and, in the case of penetrating keratoplasty, associated with the risk of devastating intra-operative complications such as suprachoroidal haemorrhage. Post-operatively, corneal grafts can fail, reject, or develop keratitis, graft failure, graft ectasia, and post-keratoplasty glaucoma. If a corneal graft fails, it can be repeated, although subsequent grafts are associated with progressively shorter survival [19,20,21]. This is a particularly pertinent consideration for those with keratoconus, because corneal grafts are frequently performed at a younger age in keratoconus than for other corneal diseases [22]. In contrast, ICRS insertion is much less invasive and has the advantage of being reversible [23]. If successful in rehabilitating vision, ICRS insertion may delay or prevent the need for a corneal transplant.

Arguably, the greatest challenge confronting surgeons when implanting corneal rings segments is determining which ICRS will induce the desired change in corneal shape in a given eye. This has led to the development of various nomograms which aim to assist surgeons in selecting the correct ring segment for a given eye. The Ferrara nomogram is one such tool. The goal of the present study was to describe the results of Ferrara ring implantation with the aid of the Ferrara nomogram in a group previously unstudied, namely, eyes with a history of cross-linking.

In the present cohort, significant improvements in visual, refractive, and tomographic parameters were found following Ferrara ring insertion. To date, the largest study examining the outcomes of Ferrara rings using the Ferrara nomogram was conducted by Lyra et al.; however, patients with a history of previous ocular surgery, including cross-linking, were excluded [13]. In the Lyra et al. study, the percentage of eyes with a CDVA of 20/40 improved from 38% pre-operatively to 90% post-operatively. This is in contrast with the present study, in which the number of eyes achieving 20/40 CDVA increased from the same baseline of 38% to only 67%. Furthermore, Lyra and colleagues found that 82% of eyes gained CDVA, with 59% of eyes gaining three or more lines. Once again, this improvement is superior to those of the present study in which only 52% of eyes gained CDVA, with 38% gaining three or more lines of CDVA. Although always difficult to compare results between two separate studies, the superior outcomes in the Lyra et al. study do raise the possibility that the difference in outcomes may be, at least in part, due to the presence or absence of prior cross-linking. This would certainly be consistent with a study by Coskunseven et al. in which the investigators found that ICRS insertion resulted in a greater improvement in vision in eyes that had not undergone cross-linking when compared to previously cross-linked eyes [14]. Certainly, further studies are warranted to compare the efficacy of the Ferrara nomogram in cross-linked compared to non-cross-linked eyes. If it is found that eyes with a history of cross-linking have poorer results than virgin corneas, this may suggest the need for the Ferrara nomogram to consider the cross-linking status of the cornea when determining which ICRS should be implanted.

HOA can be significant in determining the quality of vision in keratoconic eyes. Patients in this cohort achieved a significant reduction in the total HOA RMS from 3.65 ± 1.52 μm to 2.96 ± 1.42 μm. A recent study by Greenstein et al. investigated the change in HOA in 158 keratoconic eyes that underwent cross-linking and Intacs ICRS insertion and found an overall reduction in total HOA RMS from 4.44 ± 2.17 μm to 3.39 ± 1.94 μm [24]. The reduction in HOA is a significant finding because it enables insight into of the potential of ICRS to further reduce the HOA which is already minimized by the effect of cross-linking itself [25].

One limitation of this study is its retrospective nature and the lack of a control group. The history of prior cross-linking in these eyes is also a potential limitation, because cross-linking can result in ongoing corneal flattening with time [26]. Specific analyses based on the relationship between the exact time elapsed from cross-linking to the time of ICRS insertion were not performed. However, given that in all cases cross-linking had been performed at least 6 months prior and that the mean follow-up in this study was 4.1 months post-ICRS insertion, it is reasonable to suggest that the vast majority of the effects seen in this study were as a result of ICRS insertion. Further studies with the inclusion of corneal biomechanics measurements may assist with the understanding of the effect of cross-linking on ICRS outcomes.

In conclusion, this study is, to the best of the authors’ knowledge, the first to examine the outcomes of Ferrara ring insertion with the aid of the Ferrara nomogram in keratoconic eyes with a history of corneal cross-linking. In this series, significant improvements in visual, refractive, and tomographic parameters were found. Given that visual improvement was less than that found in the study by Lyra et al., which excluded eyes with a prior history of cross-linking, it is possible that cross-linking may lessen the effect of subsequent corneal ring implantation. If this is the case, then the authors would suggest that the Ferrara nomogram considers the presence or absence of prior cross-linking in its algorithm. Further studies would help to further clarify this hypothesis.

## Figures and Tables

**Table 1:** Baseline demographics of 21 keratoconic eyes implanted with Ferrara intrastromal corneal ring segments.

Total Number of Eyes	21
Total number of patients	19
Mean age (SD)	27.8 (7.23)
Age range (years)	17–39
Male	15
Female	4
Grade 1 keratoconus	13 (62%)
Grade 2 keratoconus	3 (14%)
Grade 3 keratoconus	3 (14%)
Grade 4 keratoconus	2 (10%)

**Table 2:** Visual, refractive, and tomographic outcomes of 21 keratoconic eyes implanted with Ferrara intrastromal corneal ring segments.

Parameter	Pre-Operative	Post-Operative	*p*-Value
Mean UDVA (SD) logMAR	0.88 (0.39)	0.52 (0.30)	<0.001
Mean CDVA(SD) logMAR	0.47 (0.32)	0.36 (0.32)	0.042
Mean SE (SD)	−7.51(3.85)	−3.76 (3.54)	<0.001
Mean Refractive Cylinder (SD)	5.72 (2.59)	3.62 (2.39)	<0.001
Mean Keratometric Astigmatism (D)	5.14 (2.91)	2.76 (1.67)	0.004
Anterior Mean K_M_	50.40 (3.53)	48.24 (3.00)	0.010
Mean K_MAX_	60.45 (6.31)	57.09 (4.92)	0.018
Mean Q value	−0.99 (0.41)	−0.67 (0.37)	0.005
Mean Corneal HOA RMS (Pentacam)	3.65 (1.52)	2.96 (1.42)	0.048

**Table 3:** Corneal tomographic changes in 21 keratoconic eyes implanted with Ferrara intrastromal corneal ring segments.

Parameter	Pre-Operative	Post-Operative	*p*-Value
**Anterior surface**			
Mean K1	47.99 (3.28)	46.93 (2.80)	0.33
Mean K2	53.14 (4.42)	49.69 (3.45)	0.008
Mean K_M_	50.39 (3.53)	48.24 (3.00)	0.01
Mean Astig	5.14 (2.91)	2.76 (1.67)	0.004
Mean K_MAX_	60.45 (6.31)	57.09 (4.92)	0.018
**Posterior surface**			
Mean K1	−7.01 (0.70)	−7.17 (0.62)	0.37
Mean K2	−8.07 (0.73)	−7.82 (0.67)	0.24
Mean K_M_	−7.49 (0.65)	−7.48 (0.60)	0.98
Mean Astig	1.05 (0.65)	0.59 (0.47)	0.005
Pachymetry thinnest	443.23 (37.26)	451 (39.76)	0.34
Pachymetry apex	458.14 (36.91)	462.6 (36.71)	0.53

## Data Availability

The dataset used in this study is privately stored by the investigators. Requests for data can be made to the corresponding author for consideration.
